# Quantitative Comparison of Tumor Delivery for Multiple Targeted Nanoparticles Simultaneously by Multiplex ICP-MS

**DOI:** 10.1038/srep05840

**Published:** 2014-07-28

**Authors:** Andrew Elias, Samuel H. Crayton, Robert Warden-Rothman, Andrew Tsourkas

**Affiliations:** 1 Department of Bioengineering, School of Engineering and Applied Sciences, University of Pennsylvania, Philadelphia PA, 19104 USA; 2 These authors contributed equally to this work.

## Abstract

Given the rapidly expanding library of disease biomarkers and targeting agents, the number of unique targeted nanoparticles is growing exponentially. The high variability and expense of animal testing often makes it unfeasible to examine this large number of nanoparticles *in vivo*. This often leads to the investigation of a single formulation that performed best *in vitro*. However, nanoparticle performance *in vivo* depends on many variables, many of which cannot be adequately assessed with cell-based assays. To address this issue, we developed a lanthanide-doped nanoparticle method that allows quantitative comparison of multiple targeted nanoparticles simultaneously. Specifically, superparamagnetic iron oxide (SPIO) nanoparticles with different targeting ligands were created, each with a unique lanthanide dopant. Following the simultaneous injection of the various SPIO compositions into tumor-bearing mice, inductively coupled plasma mass spectroscopy was used to quantitatively and orthogonally assess the concentration of each SPIO composition in serial blood and resected tumor samples.

Rapid advancements in nanotechnology have resulted in the development of nanoparticle formulations for a myriad of biological applications extending from cell tracking to improved delivery of therapeutic agents. Given the limitless ability to modify the physicochemical properties of nanoparticles to fit specific areas of interest, it is expected that their utility will only continue to increase. Recently, there has been especially significant growth in the application of nanoparticles to cancer diagnostics and drug delivery. This growth is a direct result of the numerous advantages that nanoparticles provide to this field; including, but not limited to: the ability of nanoparticles to extravasate at a tumor site, the high therapeutic and diagnostic “payloads” that can be incorporated into nanoparticles, favorable toxicity profiles, and desirable pharmacokinetic profiles that can be further manipulated by altering physicochemical properties^1^,^2^,^3^,^4^.

So far, the majority of oncology based clinical trials for nanoparticles have focused on passive delivery to tumors. That is, a nanoparticle's physicochemical properties are optimized for long blood residence time, which allows for uptake into tumors via the enhanced permeability and retention (EPR) effect^5^,^6^,^7^. While this strategy has demonstrated improved efficacy and reduced off target side-effects for nanoparticle-encapsulated therapeutics, there is increasing focus on further improving the precise delivery of these nanoparticles with active targeting strategies that utilize small molecule and biologic targeting agents. Indeed, many studies have shown that active targeting of nanoparticles can increase the dose of therapeutic delivered to a tumor and also improve the cellular uptake of delivered nanoparticles^8^,^9^. Importantly, the attractiveness of targeted platforms has recently translated to the clinic, with several targeted nanoparticles in early stage clinical assessment^10^,^11^,^12^.

Actively targeted nanoparticles offer several distinct advantages over passively targeted nanoparticles, including increased specificity for targets of interests, increased rates of internalization, and ultimately improved therapeutic efficacy and/or image contrast^13^,^14^,^15^,^16^,^17^. Despite these advantages, selection of the optimal target and targeting ligand can be difficult. Often pathologies present with a variety of known biomarkers that may be viable targets. For example, breast cancers may overexpress the estrogen receptor, progesterone receptor, and/or the Her2/neu (ErbB2) receptor^18^. As nanoparticles continue to progress toward greater clinical use, it is important to identify which molecular targets result in the best *in vivo* tumor delivery. Perhaps a more difficult problem is determining which targeting ligand is best suited *in vivo*. Importantly, the optimal molecular target and nanoparticle composition for nanoparticle delivery *in vivo* may not be accurately reflected in assays conducted *in vitro*. For example, it has been shown that affinity ligands with very high affinity do not necessarily result in the best tumor targeting, since tight binding at the tumor periphery slows diffusion of the agent within the tumor and can block extravasation of additional agent^19^. Additionally, targeting ligands can alter the physicochemical properties of a nanoparticle and the way in which it interacts with serum proteins or other off-target entities, altering the nanoparticle's pharmacokinetics and its ability to reach a tumor^20^,^21^,^22^. Importantly, it may be difficult or impossible to predict these effects prior to *in vivo* studies.

The generally accepted paradigm uses *in vitro* data to select the identity of the active targeting ligand, the ligand surface density, and other nanoparticle physicochemical properties. Subsequently, only this “optimal” formulation is transitioned to high-cost *in vivo* evaluation. However, given the large potential for incongruity between nanoparticle performance *in vitro* and *in vivo*, this approach may potentially abandon the ultimately superior formulation too early in the optimization process. Furthermore, the chosen nanoparticle is usually evaluated in one cohort of animals, while negative control (i.e. non-targeted) nanoparticle formulations are examined in other cohorts. But when nanoparticle performance is compared between separate animal cohorts, the large animal-to-animal variability characteristic of *in vivo* studies makes it more difficult to observe and assess the effect of active targeting.

The lack of optimization at the *in vivo* stage stems from several factors, including costs, the need for large animal cohorts, and the lack of a feasible “higher throughput” method for accurately comparing different nanoparticles *in vivo*. Previously, we developed a non-radiative, quantitative, and multiplex capable method for assessing nanoparticle pharmacokinetics and biodistribution, demonstrating its ability to compare passive delivery for a wide range of nanoparticle types and physicochemical properties^23^. Briefly, unique lanthanide metal dopants were used as tracers to allow for the simultaneous administration of multiple agents in a single injection, followed by downstream spectral separation of each species in blood, tissues, and tumors via inductively coupled plasma mass spectroscopy (ICP-MS). This approach is analogous to lanthanide and ICP-MS based approaches that have recently been developed for massively-multiplexed flow cytometry^24^. This system provides several advantages for comparing nanoparticle platforms *in vivo*, including significantly reduced animal cohort sizes, reduced costs, and more powerful statistical comparisons via use of a paired t-test. The potential to parse critical data such as optimal pharmacokinetics, tumor accumulation, and biodistribution for multiple targeted samples in the same cohort could allow for the holistic optimization of a nanoparticle platform *in vivo*, instead of the aforementioned compartmentalized approach to nanoparticle development.

For this work, we have extended our nanoparticle ICP-MS multiplex method to targeted nanoparticle agents. Specifically, we have selected three targets of interest: the HER2/neu receptor, heat shock protein 47 (HSP47) and α_V_β_3_ integrin, each found to be expressed within a single cancer cell line. Each of these receptors has been shown to have a high association with a wide range of cancers, and each has been used as a target in previous therapeutic studies^25^,^26^,^27^,^28^. Additionally, each of these targets has ligands that can be used to actively target SPIO nanoparticles. Specifically, HER2 affibody, cyclic RGD, and the LDS affinity peptide (Table 1) were selected as ligands for targeting HER2/neu, αvβ_3_ integrin, and HSP47 respectively^29^,^30^,^31^,^32^,^33^,^34^. A set of four lanthanide-doped SPIO nanoparticles (Ho, Sm, Gd, and Er) were synthesized. HER2 affibody, cyclic RGD, and LDS peptide were conjugated to the Ho-, Sm-, and Gd-SPIO, respectively. The Er-SPIO lacked a targeting ligand and served as a negative non-targeted control nanoparticle formulation. These constructs were first assessed for their tumor targeting capabilities *in vitro*, and then ICP-MS multiplex analysis was used to track all formulations simultaneously in single animals, with assessment of pharmacokinetics and tumor accumulation.

## Results

### Nanoparticle physico-chemical characterization

**Figure 1:**
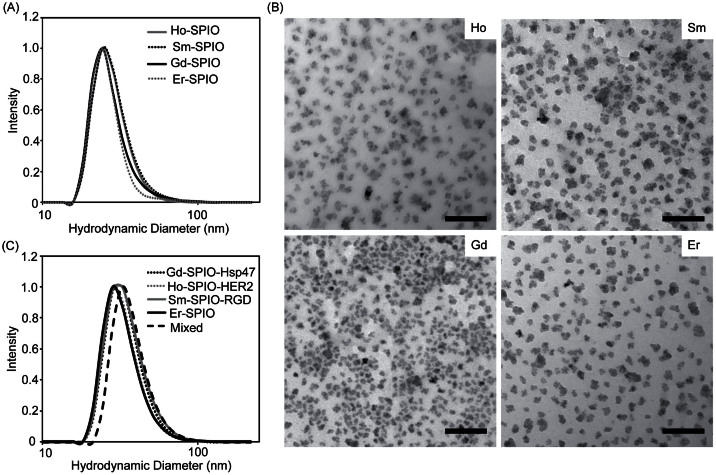
Dynamic light scattering (DLS) size distributions for Ln–SPIO nanoparticles. (A) Size comparison of the four nanoparticle formulations prior to conjugation to targeting ligands. (B) Representative TEM images of the four nanoparticle formulations. (C) Dynamic light scattering profiles of each nanoparticle formulation after conjugation to its respective targeting ligand. The size profile was also examined in a sample where all formulations were combined into a single sample (mixed).

A set of four lanthanide-doped SPIO nanoparticles (Ho, Sm, Gd, and Er) were synthesized and their physico-chemical properties were determined. Since variation in the physico-chemical properties of a nanoparticle can alter its pharmacokinetics and biodistribution, it was important to ensure that the four Ln-SPIO formulations exhibited very similar size profiles prior to targeting ligand conjugation. The hydrodynamic diameter of each Ln-SPIO formulation was determined by DLS prior to conjugation of active targeting ligands. It was found that the peak of the distribution lay between 27.00 nm and 29.07 nm for all four formulations (Table 2). Furthermore, the size distributions have a very high degree of overlap (Figure 1A) and the Ln-doped iron oxide cores are of similar size and morphology, as determined by TEM (Figure 1B), suggesting that the “base” nanoparticles to which the active targeting ligands were attached are very similar. The hydrodynamic diameter of each formulation was subsequently rechecked after conjugation of active targeting ligands (Table 2 and Figure 1C). It was found that each formulation increased in size by approximately 5 nm, so that the post-conjugation sizes ranged from 33.54 to 35.57. It is likely that the increase in size is due to the addition of the various functional groups required for conjugation (*i.e.* aza dibenzocyclooctyne^35^, linker peptide, and targeting ligand itself). Again, as before ligand conjugation, the size profiles showed a very high degree of overlap, indicating the populations are very similar in size. Thus, for the actively targeted agents, it is unlikely that any observed difference in nanoparticle pharmacokinetics or biodistribution is the result of size alterations secondary to conjugation.

For the ICP-MS multiplex method it is critical that the co-injected nanoparticles do not associate or aggregate with one another prior to injection. To this end, DLS measurements were used to rule out the possibility of nanoparticle aggregation. Specifically, all four Ln-SPIO formulations (post-conjugation) were mixed together in equal amounts and allowed to incubate together for one hour. The DLS profile of the mixed solution was then acquired (Figure 1B). Since the peak size for the mixed sample was 38.15 nm and the distribution was very similar to that of each individual formulation, it was concluded that no significant association or aggregation occurs between the actively targeted formulations prior to injection.

The zeta potential (surface charge) of a nanoparticle formulation also plays a significant role in the pharmacokinetics and biodistribution of nanoparticle platforms^36^,^37^,^38^. Therefore, the zeta potential of each Ln-SPIO was determined both before and after conjugation with active targeting ligands. For the “base” nanoparticles, the aminated nanoparticles (which would display a positive surface charge) were first carboxylated using succinic anhydride in order to generate a negatively charged surface suitable for in vivo testing. It was found that the carboxylated “base” nanoparticles had zeta potentials ranging from −4.47 mV to −6.09 mV, which were considered to be very close in value. A slightly larger degree of surface charge variation was observed in the nanoparticles after conjugation, −6.48 mV to −10.53 (Table 2). This is a reasonable expectation, since a number of factors influence what the final charge will be (*e.g.* percentage of amino groups that have undergone conjugation, the percent of remaining groups that were carboxylated, and the inherent charge of the targeting ligands). It is possible that these differences in nanoparticle surface charge may influence the formulations' blood circulation times, and consequently their tumor delivery. However, since this variation in surface charge was introduced through the process of conjugation, it falls within the realm of what we desire to test: how does the presence of active targeting ligand affect each nanoparticle's pharmacokinetics and biodistribution.

The longitudinal and transverse relaxivities of each Ln-SPIO formulation prior to ligand conjugation were also determined (Table 2). There is significant variation in the magnetic properties for the four Ln-SPIO formulations, which is not unexpected since the batch-to-batch variation in magnetic properties can be significant for traditional dextran SPIO without lanthanide dopant. While it is important to know the r2 value for each Ln-SPIO in order to normalize its MR signal during *in vitro* cell association assays, agreement between R2 values is not necessary, since MR imaging is not a primary goal of this investigation. Nevertheless, it is noteworthy that each Ln-SPIO formulation has significant magnetic activity. This is helpful since it means that once a set of nanoparticles is investigated using the ICP-MS multiplex approach, and a particular formulation that results in greatest tumor delivery has been identified, that specific formulation can then be directly administered as a single injection and evaluated for its ability to generate MR contrast.

### In vivo equivalence of nanoparticle formulations prior to conjugation

**Figure 2:**
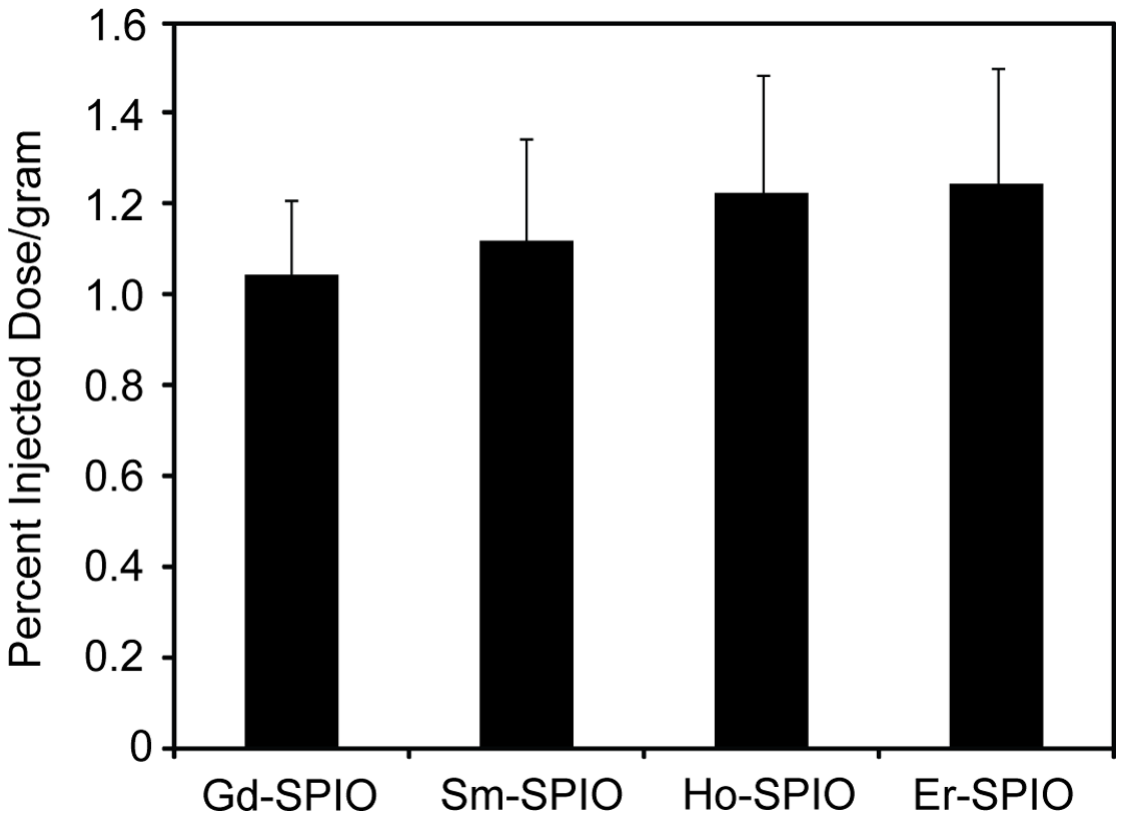
In vivo multiplex ICP-MS analysis of nanoparticle accumulation in T6-17 tumors (expressed as percent injected dose/gram of tumor tissue) for carboxylated Ln-SPIO before conjugation to active targeting ligands. ANOVA analysis yielded an F ratio of 0.594, corresponding to a P value of 0.636.

In order to conclude that differences in tumor accumulation are not due to any small differences in the physicochemical properties of the SPIO nanoparticles, it is important to demonstrate that the “base” nanoparticles, prior to ligand conjugation result in identical tumor delivery. Accordingly, each Ln-SPIO formulation was carboxylated to confer a rougly equal negative charge to all formulations (Table 2) and the set of nanoparticles was administered intravenously as a single multiplex injection to female nu/nu nude mice bearing subcutaneous T6-17 tumors (Figure 2). It was found that the tumor delivery for the four Ln-SPIO formulations ranged from 0.99 to 1.22 percent injected dose/gram of tumor tissue. One way analysis of variance (ANOVA) statistical testing demonstrated an F statistic of 0.494, corresponding to a P value of 0.594, indicating that there is no evidence of any meaningful difference in tumor delivery for any formulation within the set. Furthermore, a benefit of the simultaneous multiple injections is that the read-outs for each sample within a mouse cohort can be compared on a mouse by mouse basis by pairing statistics. We have previously shown the increased statistical power gained from this type of statistical comparison^23^. To this end, a t-test (with pairing) was conducted between the nanoparticle with lowest accumulation (Gd) and the one with highest accumulation (Er). This yielded a P value of 0.43; again suggesting that even with the improved statistical power of paired analysis, there is no significant difference between the nanoparticle formulations at “baseline”. Notably, we have previously shown that Ln-SPIO are highly stable in serum, with less than 0.5% of the lanthanide dopant being leaked upon exposure to 100% serum for 24 h at 37°C^23^. Therefore, it is unlikely that leakage of the lanthanide dopant has a significant effect on pharmacokinetic measurements. Moreover, for the dosages used, we have also previously shown that Ln-SPIO distribution is the same regardless of whether Ln-SPIO is injected individually or as a mixture^23^. Therefore, multiplexing does not seem to significantly alter Ln-SPIO pharmacokinetics or tumor accumulation.

### Assessment of biomarker expression by Western blot

**Figure 3:**
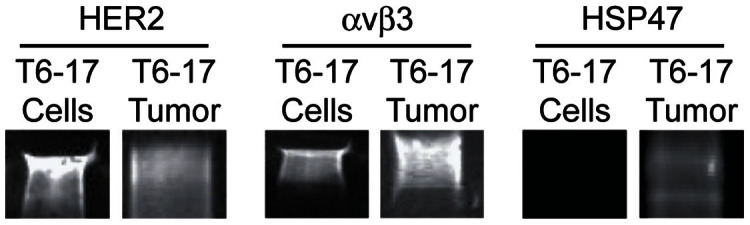
Western blot analysis of T6-17 cells and T6-17 tumor homogenates. Cells and tumor tissue were assessed for expression of (A) the HER2 receptor (B) αvβ3 integrin and (C) HSP47.

Before starting targeted studies, the expression of each biomarker (HER2/neu, αvβ_3_ integrin, and HSP47) was assessed in T6-17 immortalized cells as well as in tumor xenografts from implanted T6-17 cells. Specifically, western blots were conducted on T6-17 cells and excised, homogenized T6-17 tumors. The blot images are provided in Figure 3. Foremost, HER2/neu expression levels in T6-17 cells were high according to western blot analysis. Given that T6-17 cells are NIH-3T3 murine fibroblasts engineered to constitutively overexpress HER2, this result is expected. The relative abundance of HER2 protein in the excised T6-17 tumor appears lower than T6-17 cells *in vitro* (normalized to total protein). It is likely that the relative abundance of HER2 is lower as a result of the large amount of non-T6-17 cell derived protein in the tumor (*e.g.* stromal cells and extracellular matrix proteins). Nevertheless, high HER2 expression was still clearly evident in the excised T6-17 tumor lysate.

Next, the level of α_V_β_3_ integrin was examined. It was found that the level of expression of this biomarker was again high in T6-17 cells, although levels appear to be lower than HER2 receptor expression levels. Studies have shown integrin α_V_β_3_ expression in NIH/3T3 cells and this expression appears to be conserved in T6-17 cells^39^,^40^. Interestingly, unlike the HER2 receptor relative abundance, which drops once the entire tumor is examined, the α_V_β_3_ integrin levels are higher in the excised T6-17 tumor compared to the individual cells. This is likely because α_V_β_3_ integrin is highly overexpressed on activated endothelial cells associated with the neovascularization of tumors^41^,^42^,^43^. In fact, previous reports have shown that in tumor xenograft models α_V_β_3_ integrin can be overexpressed both on the malignant cells, themselves, and on host-derived proliferating endothelial cells^44^. This makes α_V_β_3_ integrin a particularly interesting biomarker to compare with HER2. Specifically, even though HER2 is more abundant on tumor cells than α_V_β_3_ integrin, targeting α_V_β_3_ integrin might result in increased tumor delivery, since it is expressed elsewhere in the tumor tissue. Importantly, this is a comparison that can only be adequately made *in vivo*, demonstrating the utility of being able to use ICP-MS for multiplex analysis *in vivo*.

Finally, levels of Hsp47 were examined. In this case, the expression of this biomarker was below the level of detection for T6-17 cells. Although there is little literature regarding the expression of Hsp47 on NIH/3T3 or T6-17 cell lines, it is not surprising to observe very low levels of expression since Hsp47 is most commonly associated with head and neck or gastrointestinal malignancies^25^,^27^,^45^,^46^. Interestingly, however, Hsp47 expression was clearly detectable in the excised T6-17 tumor. There are two potential possibilities to account for this observation. First, it is known that Hsp47 expression is upregulated during a cellular stress response to noxious stimuli including high temperature, heavy metal exposure, and oxidative stress^47^. Since the establishment of a rapidly growing xenograft tumor is likely to be associated with a hostile local environment, it is possible that the T6-17 cells themselves are upregulating their expression of Hsp47. Alternatively, cell populations within the tumor other than the T6-17 cells themselves may be displaying the biomarker. In either case, this again illustrates the idea that evaluating active targeting of Hsp47 directed nanoparticles is best done fully at the *in vivo* stage, since expression profiles of the tumor are not the same as those *in vitro*.

### Flow cytometric analysis of targeted Ln-SPIO

**Figure 4:**
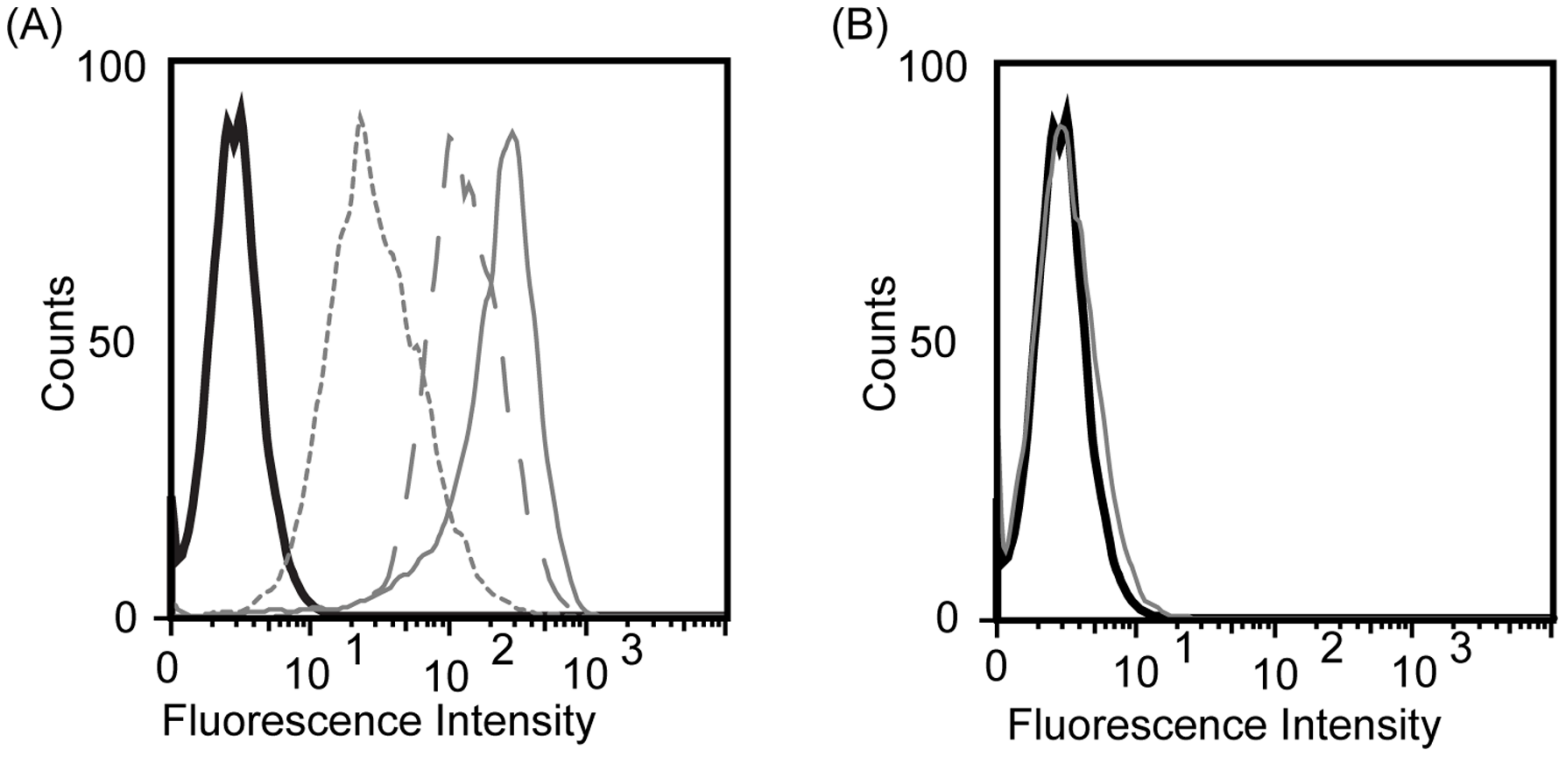
Flow cytometric analysis of T6-17 cells incubated with SPIO nanoparticles. (A) T6-17 cells were incubated with HER2-SPIO (solid gray line), RGD-SPIO (dashed gray line), and LDS-SPIO (dotted gray line), with varying degrees of cell labeling observed for each ligand. Unlabeled cells are represented by a black solid line. (B) Flow cytometric analysis of T6-17 cells incubated with each variant of the non-targeted LN-doped SPIO nanoparticles.

The functionality of HER2-SPIO, LDS-SPIO and RGD-SPIO was subsequently assessed by conducting cell-binding assays with the T6-17 cells. Flow cytometric analysis revealed that each targeted SPIO formulation successfully labeled T6-17 cells to varying extents, with the HER2-SPIO showing the highest degree of cell labeling and the LDS-SPIO showing the lowest (Figure 4 A). This is generally consistent with the results of the Western blots in that strong labeling was observed for the highly expressed HER2 receptor, and a lower level of labeling was observed for the less highly expressed α_V_β_3_ integrin. Although Hsp47 expression was not detectable on Western blots of T6-17 cells, flow analysis is likely to be more sensitive given that each nanoparticle carries multiple fluorophores, thereby amplifying the signal. As a control, Er-SPIO nanoparticles that have been reacted with ADIBO and carboxylated with succinic anhydride, but have no targeting ligand conjugated to them, showed no cell binding when incubated with T6-17 cells, thus indicating that cellular binding is not a product of the ADIBO or carboxyl surface groups on the SPIO nanoparticles (Figure 4 B).

### MR comparison of cell binding

**Figure 5:**
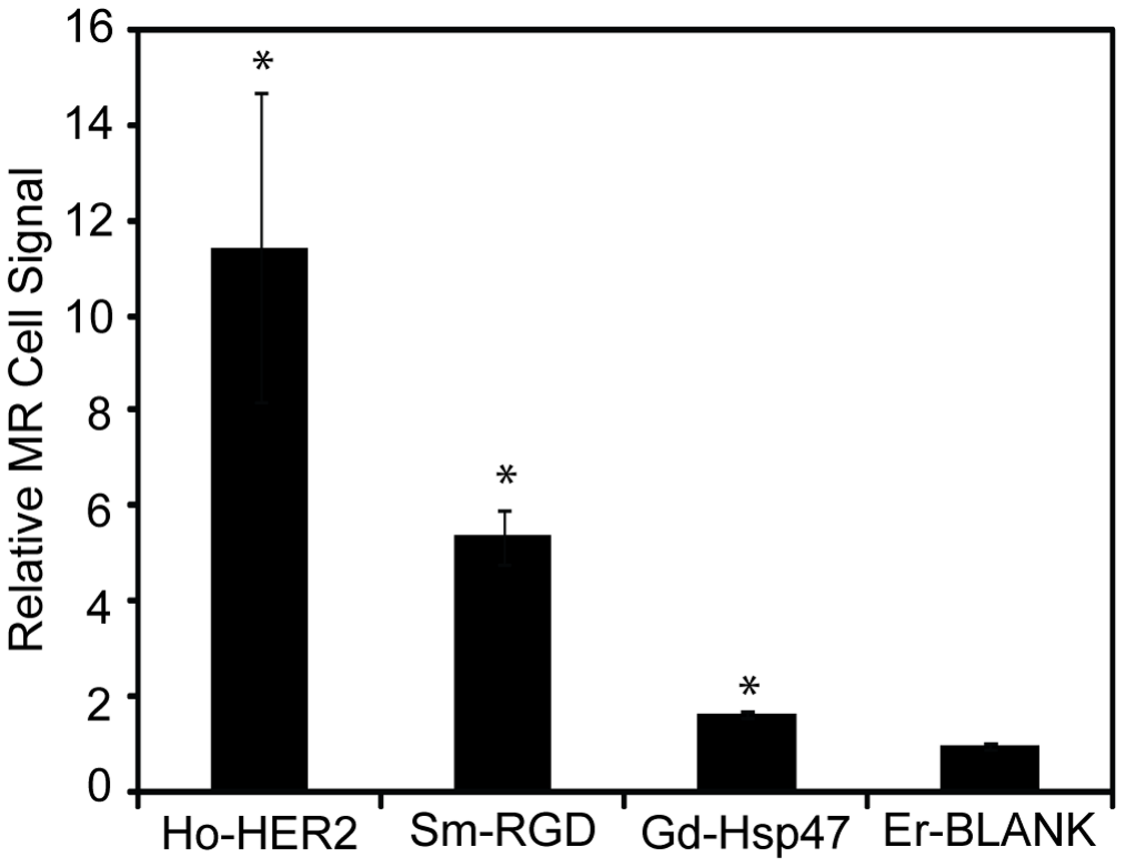
Labeling of T6-17 and HeLa cells with Ho-HER2-SPIO, Sm-RGD-SPIO, Gd-LDS-SPIO and Er-blank-SPIO, as assessed by MR relaxometry. Since each Ln-SPIO has different magnetic relaxivity (r2), the R2 relaxation signal obtained for each cell pellet was normalized by the relaxivity of the SPIO formulation and reported as a relative value to the signal of the blank formulation. All three samples exhibited statistically significant (p < 0.01) improvements in cell uptake as compared to the non-targeted control.

*In vitro* cell binding assays were also carried out by incubating targeted SPIO conjugates with T6-17 cells for 1 hour at a final concentration of 75 μg Fe/mL and examining the T2 relaxivity of the cell pellets. This assay provides a more reliable measurement for the comparison of cell binding between ligands than flow cytometry, since the fluorescence signal per nanoparticle is not expected to be the same for each formulation. For the MR assay, comparison of the level of cell labeling was made by using the reciprocal of the T2 relaxation time of the cell pellet as a measure of MR signal. The signal was adjusted by the r2 of the particular Ln-SPIO formulation used (*e.g.* Ho-SPIO for the affibody) to normalize for differences in relaxivity between samples. These data follow the same general trend as observed with the flow cytometric analysis. HER2 affibody conjugated SPIO exhibit an extremely high level of cell labeling on T6-17 cells (Figure 5). Again, it was not surprising that the HER2-SPIO displayed the highest degree of cell binding, since T6-17 cells have been transfected to overexpress the HER2/neu receptor^48^. RGD-SPIO exhibited approximately half the level of cell labeling (compared to HER2-SPIO) on T6-17 cells, but the level of labeling is clearly well above baseline nonspecific interactions observed with blank-SPIO. Finally, the T6-17cell line exhibited very low level labeling with LDS-SPIO, although even this low level of cell binding can be distinguished from the nonspecific binding of blank-SPIO.

### ICP-MS comparison of cell binding

**Figure 6:**
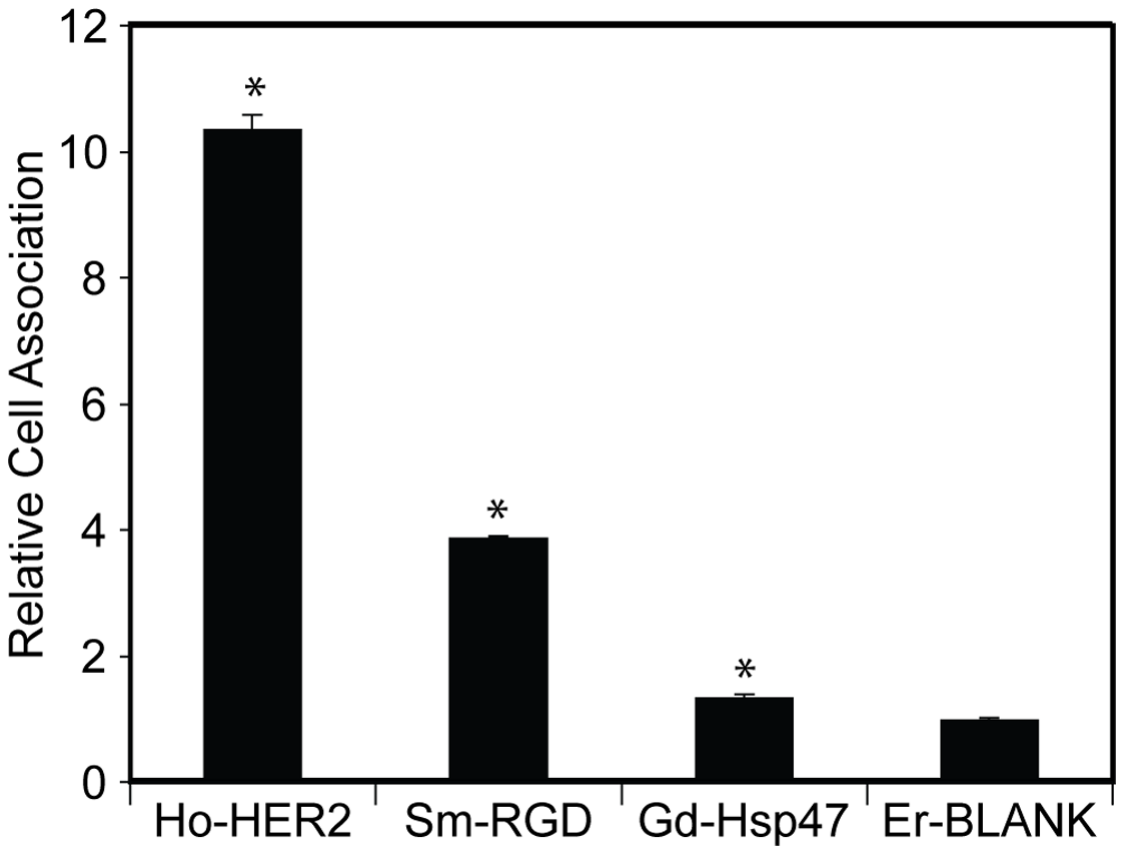
Labeling of T6-17 cells with Ho-HER2-SPIO, Sm-RGD-SPIO, Gd-LDS-SPIO and Er-blank-SPIO, as assessed by ICP-MS multiplex analysis. All targeted nanoparticle formulations were pooled together and incubated with T6-17 cells in the presence of serum supplemented culture medium. Cell uptake was calculated as a relative factor of the starting concentration of lanthanide metal in each Ln-SPIO variant and total lanthanide tracer found in cell pellets. All three samples exhibited statistically significant (p < 0.01) improvements in cell uptake as compared to the non-targeted control.

An *in vitro* ICP-MS cell binding assay was conducted by simultaneously incubating all targeted SPIO conjugates with T6-17 cells for 1 hour at a final concentration of 75 μg Fe/mL and comparing the lanthanide concentrations in the washed cell pellets versus the lanthanide concentrations in the incubating medium (Figure 6). This assay is expected to provide the most reliable data for making comparisons between ligands for several reasons. Firstly, each nanoparticle formulation's binding can be quantitatively normalized to the amount of material applied to the cells in the assay. Secondly, unlike the MR based assay, the “signal” detected by ICP-MS is linear over a very large dynamic range of nanoparticle concentrations. This is especially important at low levels of nanoparticle binding, when ICP-MS can detect differences in binding that would not translate into a difference in MR signal. Thirdly, since this assay multiplexes the measurement of cell binding, many sample-to-sample variations (such as non-specific association with dead cells) are eliminated.

The ICP-MS multiplex data again bear out the same general conclusions as the flow cytometric and MR-based assays. Foremost, with respect to T6-17 cell binding, HER2-SPIO demonstrate the greatest level of cell labeling, followed by RGD-SPIO, and LDS-SPIO, all of which are distinguishable from the non-specific blank-SPIO. Nevertheless, all three assays display differences in the levels of cell labeling observed, yet retain the general trend of HER2 > α_v_β_3_ > HSP47 in terms of targeted cell association. Regardless, the results and discrepancies between receptor expression levels and cell binding via different analytical techniques underscores the potential drawbacks of developing and optimizing targeted nanoparticle formulations based solely on *in vitro* metrics. The marriage of nanoparticle pharmacokinetics and tumor accumulation as well as tumor binding and uptake cannot be properly assessed *in vitro* and are optimally examined concomitantly *in vivo*.

### ICP-MS analysis of targeted SPIO pharmacokinetics and tumor accumulation

**Figure 7:**
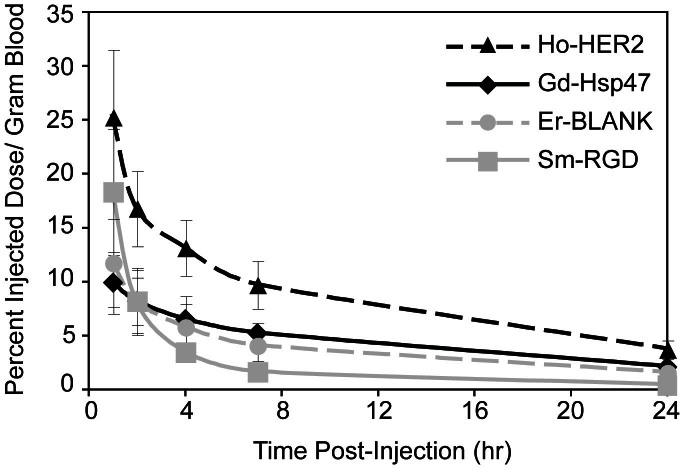
ICP-MS multiplex analysis of nanoparticle blood clearance as measured by concentration of lanthanide tracer in the blood.

**Figure 8:**
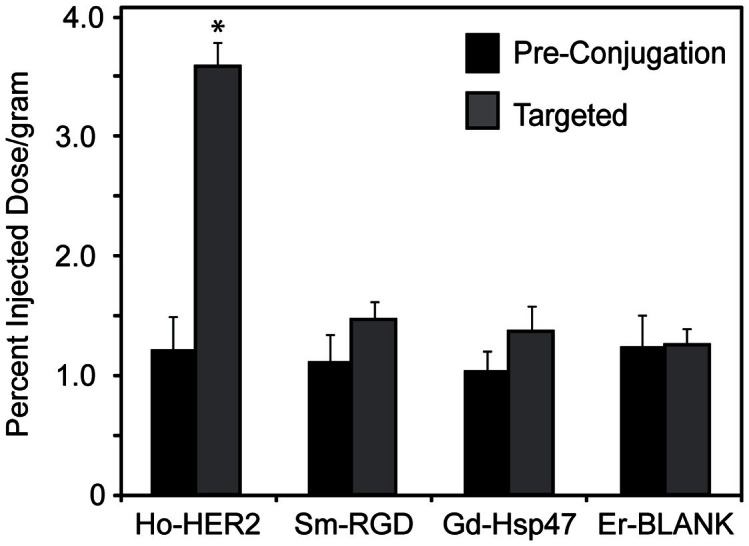
Comparison of quantitative tumor delivery of Ln-SPIO nanoparticles pre-conjugation (black) and post-conjugation (gray) to targeting ligands as determined by ICP-MS multiplex analysis. All targeted samples saw quantitative increases in tumor accumulation; however, only Ho-HER2-SPIO elicited a statistically significant increase over its parent control (p < 0.001).

The addition of targeting ligands to the surface of nanoparticles could significantly impact nanoparticle pharmacokinetics in a number of ways, including altered physicochemical properties, altered interactions with blood serum proteins and off-site accumulation of targeted nanoparticles into tissues natively expressing the targets of interest^49^,^50^. Before investigating the tumor uptake of the targeted nanoparticles, the pharmacokinetic profile of each sample was assessed at five timepoints over their first 24 hours in circulation (Figure 7). Foremost, the pharmacokinetic data of the control SPIO correlates indistinguishably with previously published multiplex data for this platform, highlighting the consistency and accuracy with which this method may be employed^23^. Moreover, tumor accumulation for the control SPIO is nearly identical when comparing pre- and post-conjugation tumor accumulation data (Figure 8). For the targeted samples, at the 1 hour timepoint blood concentration levels of both the HER2-SPIO and the RGD-SPIO are significantly higher than the LDS-SPIO (p < 0.05). The early blood concentration differences cannot be attributed to difference in the quality or success of sample administration, as all four nanoparticles were administered as one single injection. More than likely, early differences in blood concentrations stem from effects attributable to the nanoparticle's surface properties (i.e. ligands and chemical groups), which will ultimately control their interactions with serum proteins, aggregation, and redistribution in the mouse blood pool. Continued observations of blood concentrations reveal significant differences between HER2-SPIO and LDS-SPIO until the final 24 hour timepoint. Alternatively, RGD-SPIO, despite having relatively high blood concentrations at the 1-hour timepoint, undergoes an accelerated blood clearance, such that at the 7 hour timepoint LDS-SPIO has a significantly higher blood concentration (p < 0.05). The faster clearance of RGD-SPIO is not surprising, as αvβ_3_ integrin is known to be natively expressed and previous publications have also demonstrated increased clearance rates when employing RGD-targeted nanoparticles^51^,^52^.

The downstream effects of these blood concentration levels are evident when assessing the total tumor uptake for each of the nanoparticles (Figure 8). First, while all of the targeted nanoparticles displayed a quantitative increase in tumor accumulation over the non-targeted controls from Figure 2, only the HER2-SPIO demonstrated a statistically significant difference both between the non-targeted control (p < 0.01) and also the other targeted nanoparticles (p < 0.01). It is possible that the improved tumor accumulation can be at least partially attributed to the increased circulation concentrations of the HER2-SPIO. The reason for the increase circulation concentrations is not directly apparent when looking at the physicochemical parameters of the HER2-SPIO as compared to the other nanoparticle variants. All formulations had similar sizes and surface charges in their final injection state. Additionally, the Er-blank non-targeted SPIO nanoparticles had indistinguishable absolute tumor uptake in both *in vivo* studies (pre and post conjugation), highlighting the similarities between the two animal cohorts. It is possible that by surface decorating the SPIO with HER2-affibodies, the interactions of the nanoparticles with serum proteins and ultimately the mononuclear phagocyte system (MPS) has been altered in a favorable manner. This would be consistent with previous studies, which have shown that altered interactions with serum proteins or other off-target entities can influence a nanoparticle's pharmacokinetics and its ability to reach a tumor^20^,^21^,^22^. Regardless, these results underscore the premise that optimization of targeted nanoparticle formulations can benefit greatly by assessing pharmacokinetic and biodistribution *in vivo*, as it is difficult to accurately predict ultimate nanoparticle behavior and fate using commonly employed *in vitro* metrics.

## Conclusion

It is possible to synthesize SPIO nanoparticles, doped with a variety of lanthanide tracer metals, each with an overlapping size distribution, so that they exhibit equal levels of passive tumor accumulation. These Ln-SPIO formulations can then be subsequently functionalized with active targeting ligands, such that each targeting ligand is associated with a specific lanthanide tracer. ICP-MS analysis can quantify the concentration of each lanthanide metal independently and with very high sensitivity, in a single fluid or tissue sample. Therefore, it becomes feasible to collect nanoparticle blood residence time, tumor delivery, and biodistribution for many actively targeted and negative control formulations in a single animal. This represents a powerful tool for nanotechnology investigators to holistically optimize nanoparticle formulations for *in vivo* performance, while reducing experiment time, cost and number of animals.

## Methods

### Materials

Azido-dPEG_4_-NHS ester was purchased from Quanta BioDesign Ltd. (Powell, OH). NIH/3T3 cells that were engineered to stably express the Her2/neu receptor (T6-17) were kindly provided by Dr. Mark Greene, MD/PhD (University of Pennsylvania). ADIBO-dPEG_4_-NHS was kindly provided by Vladimir Popik, Ph.D. (University of Georgia). All other reagents were purchased from Thermo Fisher Scientific (Waltham, MA) unless otherwise noted.

### Synthesis of dextran stabilized lanthanide doped SPIO

Dextran coated, lanthanide doped, SPIO nanoparticles were prepared though the coprecipitation of ferrous, ferric, and lanthanide ions in the presence of dextran as described previously^53^. After the reaction, the Ln-SPIO was purified by diafiltration across a 100 kDa membrane and was 0.2 μm filtered to remove any oversized material. Finally, to ensure complete purification of the Ln-SPIO from excess salt, lanthanide ions, and FITC, the nanoparticles were magnetically purified on MACS LS columns using a MidiMACS magnet (Miltenyi Biotec, Auburn, CA, USA). Control particles, i.e. those not labeled with targeting ligands, were reacted at a 1:10 molar ratio of SPIO:FITC in pH 9 sodium bicarbonate buffer. The labeled particles were purified on a PD10 gel filtration column in PBS to yield approximately 3 dye molecules per particle.

### Cloning of HER2-Affibody and LDS recombinant protein into pTXB1 vector

The nucleotide and corresponding amino acid sequences for the HER2 affibody and LDS affinity peptide are provided in Table 1. Complementary oligonucleotides comprising the HER2-Affibody or LDS coding sequence flanked at both ends by 15 base sequences homologous to the desired restriction sites of the destination vector were ordered from Integrated DNA Technologies (**Coralville, IA)**. To improve subsequent affinity column cleavage, an additional 9 base pairs encoding a “MRM” amino acid sequence were included in the oligonucleotides at the C-terminal end of both sequences. Oligonucleotides were hybridized and the resulting sequence was ligated with *Nde*I-*Xho*I double digested pTXB1 vector (New England Biolabs, Inc) via the CloneEZ kit (Genscript). Insertion of the HER2-Affibody and LDS sequences was verified by DNA sequencing using the T7 promoter as the sequencing primer.

The pTXB1-HER2-Affibody vector was transformed in Rosetta™ 2(DE3)pLysS Competent Cells (Novagen). Following induction of protein expression with IPTG (0.5 mM), cells were lysed and the affibody was affinity purified using a Poly-Prep chromatography column (Bio-Rad, Hercules, CA) packed with 1 mL of chitin beads (New England Biolabs, Inc). Supernatant was allowed to pass through the column and chitin beads were washed with 50 mL of column buffer at a flow rate of approximately 2 mL/min. Three mL of 50 mM MESNA was quickly passed through the column in order to evenly distribute the MESNA throughout the chitin beads, and flow was stopped. The column was incubated for 16 hours at 4°C. HER2-Affibody proteins, now containing a C-terminal thioester, were eluted from the column in a total 4 mL buffer (0.1 M Tris-HCl, pH 8.5) and concentrated to a volume of 500 μL using an Ultracell 3,000 (Millipore, Billerica, MA). An analogous experimental protocol was used for the production and purification of LDS peptides, with the exception of the IPTG concentrations used for induction, which were lowered from 0.5 mM to 0.4 mM final concentration.

### Expressed protein ligation

Expressed protein ligation was carried about between the thioester containing HER2-Affibody/LDS peptide and an azido-fluorescent peptide (AzFP) with an N-terminal cysteine. The sequence of the AzFP was NH_2_-CDPEK(5-FAM)DSGK(N3)S-OH. The K(5-FAM) represents a lysine with a fluorescein covalently attached to its ε-amino group and the K(N3) represents a lysine with an azido group attached to its ε-amino group. The AzFP (0.1 mM) was incubated with approximately 0.01 mM HER2-Affibody or LDS. The EPL reaction was mixed overnight at room temperature. For the HER2-Affibody, the EPL product and excess AzFPs were separated on a Superdex 30 chromatography column. For the LDS-peptide, several rounds of washing using Ultracell 3,000 filtration columns were used to remove unreacted AzFP peptides.

### Azide functionalization of Cyclic-RGD

Cyclic-RGD (Anaspec, Fremont, CA; Table 1) was incubated with Azido-dPEG12-NHS at 10:1 molar ratios of Azide:RGD in DMSO at a final volume of 30 μL. Reactions were incubated at room temperature overnight and purified via RGD precipitation in 10× volumes of tert-butyl methyl ether followed by centrifugation at 16,000× g for 1 minute. These precipitations were performed in triplicate and the resulting conjugate was suspended in a final volume of 30 μL DMSO.

### ADIBO modification of SPIO NPs for click chemistry

Surface amines on SPIO NPs were reacted with the amine-reactive ADIBO-dPEG_4_-NHS in 0.1 M sodium phosphate buffer, pH 9. ADIBO is an alkyne-containing moiety suitable for copper-free click conjugation to the azide-containing ligand preparations^35^. Specifically, a 138 mM stock of ADIBO-dPEG_4_-NHS was diluted 100-fold into a 50 μM solution of SPIO NPs. All nanoparticle solutions were mixed overnight at room temperature. SPIO NPs were purified via superdex 200 chromatography columns (GE Healthcare, Piscataway, NJ). The resulting ADIBO-SPIO NPs were incubated with 100 times molar excess of succinic anhydride to convert all remaining amines to carboxyl groups. ADIBO-SPIO NPs were subsequently purified on superdex200 chromatography columns, equilibrated with PBS. For RGD-SPIO and unlabeled SPIO used in flow cytometry experiments, SPIO NPs were first labeled with a FITC fluorophore (10:1 molar ratio of FITC:SPIO) and purified via PD-10 purification columns before being labeled with ADIBO.

### Copper-free click conjugation

ADIBO-SPIO NPs (1 mg/mL) were mixed with fixed concentrations of HER2-AzFP ligand (20 μM) and LDS-AzFP (30 μM) in PBS, pH 7.4 at a final volume of 100 μL. For RGD-N3, 60 μM of the peptide was incubated with ADIBO-SPIO NPs (1 mg/mL) in a final volume of 100 μL. Reactions were mixed overnight at room temperature and then purified on Superdex 200 chromatography columns equilibrated with PBS.

### Nanoparticle physicochemical characterization

Stock samples of Ln-SPIO nanoparticles were diluted into pH 7.4 phosphate buffered saline for determination of the hydrodynamic diameter by dynamic light scattering (DLS) both before and after conjugation to active targeting ligands. Measurements were acquired with a Zetasizer Nano-ZS (Malvern Instruments, Worcestershire, UK) using the non-invasive back-scatter (NIBS) mode. For zeta potential measurements, stock samples of Ln-SPIO were diluted into phosphate buffered saline at pH 7.4 and the mean nanoparticle zeta potential was measured, both before and after conjugation to targeting ligands, using a Zetasizer Nano-ZS. For Ln-SPIO nanoparticles, the transverse (r_2_) and longitudinal (r_1_) relaxivities were measured using a Bruker mq60 tabletop MR relaxometer operating at 1.41 T (60 MHz).

### Cell culture

T6-17 murine fibroblasts (a derivative of the NIH/3T3 line and kindly provided by Mark Greene, PhD, FRCP, University of Pennsylvania) were cultured and maintained in Dulbecco's modified Eagle's medium (DMEM), supplemented with 10% fetal bovine serum (FBS), 1% penicillin/streptomycin at 37°C and 5% CO_2_.

### Western blots

T6-17 cells were grown to 80% confluence on 10 cm plates. The plates were washed twice with PBS and then incubated on ice for five minutes in 1 mL RIPA Buffer (Sigma-Aldrich) supplemented with 6 M urea. Cells were scraped off the plate and clarified by centrifugation. 47 mg of solid tumor was solubilized in 3 mL Western Lysis Buffer (12.5 mM Tris, 4% SDS, pH 8) with a mortar and pestle. Lysate was boiled for 30 min and clarified by centrifugation. Total protein concentrations were determined by BCA Assay (Pierce). Hsp47, integrin, and ErbB2 were analyzed by Western blot. Blots were incubated for 1 hr with Odyssey Blocking Buffer (Li-Cor), washed 3 times with TBS-T, stained with antibodies against Hsp47, Integrin αV, and ErbB2 (AbCam ab109117, ab16821, and ab8054) overnight at 4C, washed 3 times with TBS-T, stained with fluorescently-labeled anti-rabbit and anti-mouse secondary antibodies (Li-Cor) for 1 hr at room temperature, washed 3 times with TBS-T, and imaged on a Li-Cor Odyssey.

### Flow cytometric analysis

T6-17 cells were dissociated from culture flasks using PBS-based enzyme free dissociation buffer and transferred to sterile 96-well plates at a final concentration of 50,000 cells per well. Targeted SPIO conjugates were added to the wells for 30 minutes at 37°C at a final concentration 75 μg Fe/mL. Cells were transferred to 1.5 mL centrifuge tubes and washed in triplicate by pelleting cells at 1000 RCF for 2 minutes and then resuspending in PBS. Cells were resuspended in 250 μL of PBS and transferred to a 96-well plate (50,000 cells per well) and analyzed using a Guava Easycyte Plus system (Guava Technologies, Hayward, CA). Flow cytometry data were analyzed using *FlowJo* software (TreeStar Inc., San Francisco, CA).

### Cell relaxation studies

T6-17 cells were dissociated using PBS-based enzyme free dissociation buffer and transferred to sterile 48-well plates at a concentration of 3 × 10^6^ cells per well. Actively targeted SPIO conjugates and unlabeled SPIO were incubated with these cells in the 48-well plate at a final concentration of 75 μg Fe/mL for 1 hour at 37°C (n = 3 for each targeting agent). Cells were transferred to 1.5 mL centrifuge tubes and washed in triplicate by pelleting cells at 1,000 RCF for 3 minutes and then resuspending in PBS. Cells were suspended in a final volume of 300 μL PBS and T2 measurements were taken using the benchtop relaxometer. The reciprocal of the T2 relaxation time constant (R2) was calculated, and the reciprocal of the T2 for cells incubated without nanoparticles (background) was subtracted off. Finally, since each Ln-SPIO formulation has a different r2 relaxivity value, the MR signal for each cell pellet was normalized by dividing by the R2 value of the particular Ln-SPIO used, resulting in a metric that is proportional to nanoparticle cellular association.

### In vitro ICP-MS multiplex assessment of cell labeling

T6-17 cells were dissociated and incubated with actively targeted SPIO conjugated and unlabeled SPIO in the same manner as in the cell relaxation studies, with the notable exception that all SPIO formulations were incubated together with cells, rather than each SPIO formulation being incubated separately. Following washing to remove unbound nanoparticles, the pellet was resuspended in 100 μL of PBS. The lanthanide concentration of Ho, Sm, Gd, and Er was then determined in each pellet and compared to the concentration present in the incubating medium. All values were then normalized by the ratio for the Er-blank formulation.

### In vivo studies

Approximately 6-week old female nu/nu nude mice (Charles River Laboratory, Charles River, MS, USA) were housed under USDA- and AAALAC-approved conditions with free access to food and water and maintained in accordance with the Institutional Animal Care and Use Committee (IACUC) of the University of Pennsylvania. All experiments were approved and performed in accordance with IACUC guidelines and regulations. Mice were anesthetized via isoflurane and T6-17 cells were injected subcutaneously into the back right flank (2 × 10^6^ cells in 0.2 mL PBS). Tumors were grown until the diameter was approximately 8 mm. Ln-SPIO (Ho, Gd, Sm, and Er) were pooled and injected intravenously at a dose of 3.75 mg Fe/kg body weight. Prior to injection, an aliquot was saved for ICP-MS determination of lanthanide concentration in injected material. Following nanoparticle injection, 10 μL blood samples were collected from each animal, using the tail-nick method, at times of 1, 2, 4, 7, and 24 h post-injection. After the final blood draw, the animals were sacrificed and the tumors excised. Tumor samples were thoroughly washed with phosphate buffered saline (PBS) and blotted dry to minimize the contribution of any nanoparticles still circulating in the blood at 24 h.

For ICP-MS analysis, analytical standards were purchased from SCP (Champlain, NY, USA) and trace metal grade nitric acid was purchased from Fisher Scientific (Pittsburg, PA, USA). All dilutions were done using in-house deionized water (≥18 MΩ-cm) obtained from a Millipore water purification system. The pre-injection solutions, blood, and tumor samples were analyzed for ^158^Gd (gadolinium), ^147^Sm (samarium), ^165^Ho (holmium), and ^166^Er (erbium) using an Elan 6100 ICP-MS (Perkin Elmer, Shelton, CT, USA) at the New Bolton Center Toxicology Laboratory, University of Pennsylvania, School of Veterinary Medicine, Kennett Square, PA, USA. The samples were weighed into Teflon PFA vials (Savillex, Minnetonka, MN, USA) and digested overnight with 70% nitric acid at 70°C. 0.1 mL of 2 ppm ^159^Tb (terbium) was added to each of the digested samples as an internal control and the mixtures were diluted with deionized water to a final volume of 10 mL. The lanthanide concentration of each sample was measured using a calibration curve of aqueous standards at 0.01, 0.1, 1.0, and 10 ppb for each metal.

The performance of the instrument and accuracy of the results were monitored by analyzing a reagent blank and bovine serum control serum (Sigma) prior to analysis of the samples. Also, standard reference material (Peach Leaves 1547) obtained from National Institute of Standards and Technology (NIST, Gaithersburg, MD, USA) with known values of iron and rare earth elements was analyzed with each batch of samples. For each nanoparticle formulation, the tumor delivery was calculated as a percent injected dose per gram of tissue as [Ln]_tumor_/([Ln]_inj_*M_inj_), where [Ln]_tumor_ is the lanthanide concentration in the tumor, [Ln]_inj_ is the lanthanide concentration in the injected nanoparticle solution, and M_inj_ is the mass of nanoparticle solution injected (0.2 grams). For evaluation of “base” nanoparticles prior to ligand conjugation, one way ANOVA analysis was used to assess similarity in tumor delivery for the different Ln-SPIO formulations.

## Figures and Tables

**Table 1:** Targeting ligands sequences

Targeting Ligand	Peptide Sequence
Her2 Affibody	VDNKFNKEMR NAYWEIALLP NLNNQQKRAF IRSLYDDPSQ SANLLAEAKK LNDAQAPKMR M
LDS	LDSRYSLQAA MYMRM
RGD	RGDfK

**Table 2:** Physico-chemical properties of targeted SPIO nanoparticles

Dopant	Ligand	Pre-Conjugation Size (nm)	Post-Conjugation Size (nm)	Pre Conjugation Zeta (mV)	Post Conjugation Zeta (mV)	r1 (mM^−1^s^−1^)	r2 (mM^−1^s^−1^)
**Er**	None	27.00	33.54	−5.63	−10.01	6.2	262.9
**Ho**	HER2-Affibody	28.07	33.47	−4.47	−10.53	10.3	135.2
**Sm**	RGD	27.77	35.57	−6.09	−6.48	9.2	158.5
**Gd**	LDS	29.07	34.84	−5.77	−8.61	8.1	172.6
